# Methylthiohexa‐1,3‐Diene: Anionic Polymerization of a Diene with Thioether Moiety Enables Post‐Polymerization Modification and Antimicrobial Materials

**DOI:** 10.1002/anie.202508129

**Published:** 2025-08-10

**Authors:** Moritz Rauschenbach, Matthias Bros, Holger Frey

**Affiliations:** ^1^ Department of Chemistry Johannes Gutenberg‐University Mainz Duesbergweg 10‐14 D‐55128 Mainz Germany; ^2^ University Medical Centre Johannes Gutenberg‐University Mainz Langenbeckstraße 1 D‐55101 Mainz Germany

**Keywords:** Anionic polymerization, Antimicrobial polymers, Diene polymers, Synthetic rubber

## Abstract

Anionic polymerization of 1,3‐dienes is a highly established approach for the synthesis of synthetic rubber and plays a key role for thermoplastic elastomers like poly(styrene‐*b*‐butadiene‐*b*‐styrene) (SBS). Aiming at novel concepts for tailoring the property profile of synthetic rubber in post‐polymerization reactions, we introduce the thioether‐containing 6‐methylthio‐hexa‐1,3‐diene (MTHD) as a diene monomer derived from the biobased methional. The obtained isomeric cis‐/trans‐mixture was successfully polymerized via anionic techniques. In situ ^1^H nuclear magnetic resonance (NMR) kinetics revealed different reactivities of the *trans*‐ and *cis*‐isomer and dispersities of Ɖ = 1.21–1.35. Copolymerization with isoprene afforded a series of well‐defined statistical copolymers with molar masses up to 50 kg mol^−1^, permitting precise adjustment of MTHD content from 2% to 10%. These copolymers exhibited moderate to narrow distributions (Ɖ < 1.19). Targeting the thioether groups via post‐polymerization modifications (i.e., alkoxylation and alkylation) permitted tailoring of the properties of the diene‐based copolymers. The respective sulfoxide was obtained selectively by mild oxidation and represents an internal antioxidant group. Introduction and testing of sulfonium functional polyisoprenes regarding their inhibition of the growth of gram‐negative and gram‐positive bacteria strains showed significant antimicrobial performance of the copolymers against *Escherichia coli* and *Staphylococcus aureus*.

## Graphical abstract

The thioether‐containing 6‐methylthio‐hexa‐1,3‐diene (MTHD) monomer is prepared from the biobased methional. The obtained isomeric cis‐/trans‐mixture of the diene can be successfully homo‐ and copolymerized via anionic techniques. Targeting the thioether groups via post‐polymerization modifications (i.e. alkoxylation and alkylation) permits tailoring of the properties, for example for antimicrobial materials.

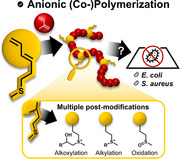

## Introduction

Antimicrobial polymers (AMPs) are intensively discussed as materials to tackle the growth of antibiotics resistant pathogens.^[^ ^1^, ^2^, ^3^, ^4^ ^]^ As indicated by the World Health Organization (WHO), this represents a significant global health concern.^[^ ^5^ ^]^ Consequently, AMPs have been rapidly developed in recent years. A widely accepted mechanism is electrostatic interaction of the anionic bacterial membrane with cationic polymers, effectively disrupting the structure and ultimately leading to bacterial cell lysis.^[^ ^3^ ^]^ Spontak et al. described a meanwhile commercially available, sulfonated pentablock copolymer. This material, which is distributed by *Kraton Corporation* under the tradename *Nexar*, inhibits the growth of a large variety of bacteria.^[^ ^4^, ^6^, ^7^, ^8^, ^9^ ^]^ Alternatively, sulfonium groups derived from thioethers introduce antimicrobial behavior with high performance against gram‐positive strains.^[^ ^1^, ^10^, ^11^, ^12^, ^13^ ^]^

In recent years, thioethers have emerged as a subject of growing interest within the field of polymer science and have been introduced in a variety of polymer classes. Their high potential was originally recognized by Deming and coworkers, who investigated click‐type reactions of thioether moieties in poly(l‐methionine) with different electrophiles.^[^ ^14^, ^15^ ^]^ This modification was systematically developed with multiple alkyl halides and epoxides, affording functional polypeptides with sulfonium groups. Several researchers as well as our group designed novel monomers inspired by l‐methionine to take advantage of the broad toolbox of suitable reagents.^[^ ^16^, ^17^, ^18^, ^19^, ^20^, ^21^ ^]^ Although the thioether moiety is a non‐protic group, modification of thioethers could induce antimicrobial behavior and enabled utilization as polymer electrolytes.^[^ ^18^, ^20^ ^]^ The oxidation to polar sulfoxides was utilized for direct tailoring of the materials properties.^[^ ^19^, ^21^, ^22^, ^23^ ^]^ This allowed for the synthesis of oxidation‐responsive, water‐soluble micelles comprising a polymer block with thioethers for drug delivery applications.^[^ ^17^, ^19^, ^21^, ^22^, ^24^ ^]^ In the field of antimicrobial materials, sulfonium groups have emerged as a promising alternative to traditional nitrogen‐based groups.^[^ ^3^ ^]^ In terms of material performance, Du Prez and coworkers presented the reversible transalkylation of sulfonium salts as a platform for the synthesis of vitrimers.^[^ ^25^, ^26^ ^]^ Despite the increasing number of thioether‐bearing monomers and applications of the resulting polymer materials, to the best of our knowledge, the use of thioether groups in the anionic polymerization of 1,3‐dienes has not been considered, although it may lead to new applications while using an industrially established polymerization technique.^[^ ^27^, ^28^ ^]^

The anionic polymerization of 1,3‐dienes is a pivotal method for the synthesis of synthetic rubber, inspired by natural rubber (*cis*‐1,4 polyisoprene).^[^ ^29^, ^30^ ^]^ Anionic polymerization offers precise control over molar mass and affords narrow distributions, leading to low vinyl content in nonpolar media.^[^ ^31^, ^32^, ^33^ ^]^ The latter is highly relevant for low glass temperatures (*T*_g_) that are important for thermoplastic elastomers. These materials are employed, for example, in medical applications that would benefit from antimicrobial properties.^[^ ^34^ ^]^

Although diene monomers have been widely investigated in materials for an extraordinary variety of commercial products, there are still limitations that could be overcome by functional moieties. Post‐polymerization reactions are currently studied to increase the polarity of polydienes, albeit with limited control over the degree of functionalization.^[^ ^35^ ^]^ Functional monomers can lead to quantitative and regiospecific modification of the polymer. However, only a limited number of functional diene monomers has been identified to date.^[^ ^33^, ^36^, ^37^, ^38^, ^39^ ^]^ The carbanionic polymerization technique is incompatible with protic, functional groups.^[^ ^40^, ^41^, ^42^ ^]^ Often, suitable protective groups must be utilized which leads to challenging monomer syntheses. On the other hand, it is necessary to implement an alkyl spacer between the 1,3‐diene moiety and the functional group to prevent the so‐called “back side collapse”. As described by Takenaka et al., the reactive center can induce an SN2‐type reaction leading to the formation of a *ω*‐isoprenyl group.^[^ ^41^, ^42^ ^]^

Recently, based on the monoterpene myrcene, functional diene‐monomers were developed that exhibited an alkyl spacer.^[^ ^37^, ^38^ ^]^ Hence, repeating units with a single or two hydroxyl groups, protected by silyl and ketal protection groups, respectively, were incorporated into the backbone of polydienes via living anionic polymerization. For instance, polymerization of a dioxolane functionalized myrcene structure was reported. In both cases, evidence for interactions of the monomer with the counterion were postulated, explaining the observed increase of 3,4‐units in the final polydiene backbone. For amino‐functional isoprene derivatives, the coordination of the nitrogen to the lithium counterion was shown by X‐ray characterization.^[^ ^39^, ^43^, ^44^ ^]^

This work reports the synthesis and anionic (co)polymerization of 6‐methylthio‐1,3‐hexadiene (MTHD) prepared from l‐methionine‐derived methional. The thioether functional polydienes were used for further polymer modification. For instance, the nucleophilicity of sulfur was targeted in a variety of post‐polymerization modifications. Modified samples from alkylation and alkoxylation reaction have been investigated with respect to their inhibition of bacterial growth of *Escherichia coli* and *Staphylococcus aureus*.

## Results and Discussion

### Monomer Synthesis MTHD

**Scheme 1:**
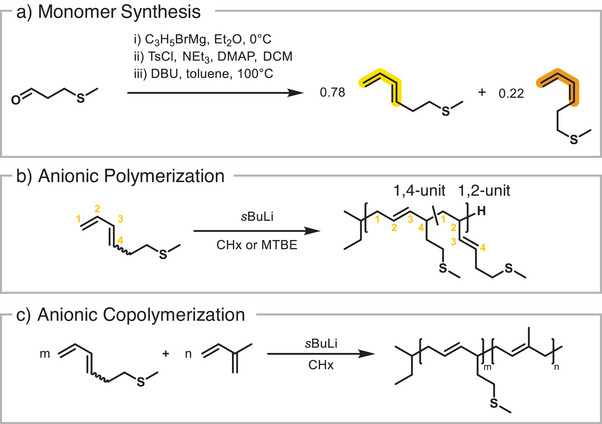
Summary of the synthesis and polymerization: a) monomer synthesis based on methional, i) Grignard reaction, ii) tosylation and iii) elimination reaction to the isomeric 1,3‐diene MTHD; b) homopolymerization of MTHD in cyclohexane or MTBE; c) copolymerization of MTHD with isoprene as a comonomer, targeting a variety of compositions. For clarity, we excluded the vinylic microstructures of isoprene and MTHD.

MTHD was synthesized in a three‐step route, starting with Grignard reaction of methional in accordance with literature procedures.^[^ ^45^ ^]^ Directly after work‐up, tosylation of the hydroxyl group with tosyl chloride and subsequent elimination of the tosylate afforded the 1,3‐diene in a DBU‐catalyzed reaction (Scheme 1a).^[^ ^46^ ^]^ The product was purified via distillation under reduced pressure. It is important to mention that this is the only purification step of this synthesis route, which is required to increase the total yield of the MTHD monomer. The product was analyzed by various NMR techniques (Figures S2–S6), revealing MTHD as an isomeric mixture, with *trans*/*cis*‐isomer ratio (^1^H NMR, Figure S2) of 78:22.

### Homopolymerization of MTHD

**Figure 1:**
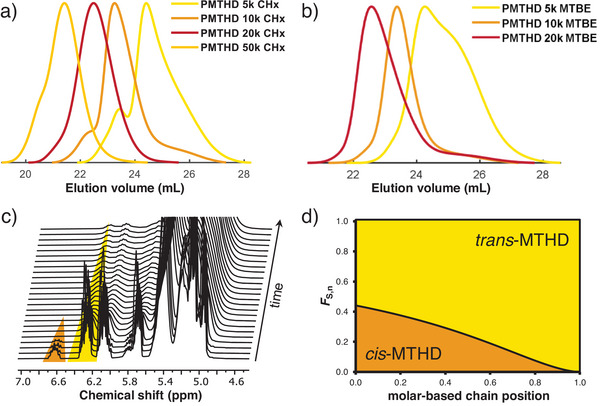
SEC traces (RI Signals, eluent: THF) of PMTHD polymerized a) in CHx or b) in MTBE. c) Zoomed‐in region of stacked spectra as a function of time. The highlighted proton signals were used to study the reaction of *trans*‐ and *cis*‐MTHD, respectively in C_6_D_12_ at 20 °C; d) simulated molar composition profile based on the reactivity ratios with the obtained ratio of both isomers after the synthesis.

Homopolymerization of MTHD was conducted using established carbanionic conditions (Scheme 1b).^[^ ^47^ ^]^ The initial experiments were performed using the non‐polar solvent cyclohexane at room temperature. A series of PMTHD polymers with molar masses ranging from 5 to 50 kg mol^−1^ were targeted. Upon initiation, the living polymer solutions turned pale yellow. The color persisted until termination, indicative of the living carbanionic chain end. Noteworthy, samples with targeted molar masses >10 kg mol^−1^ turned turbid shortly after initiation, suggesting poor solubility of the homopolymer. The results in Table 1 indicate good control over molar mass, but dispersities are considerably higher than typical for living polymerizations with values reaching 1.35. As reported for ocimene, the use of an isomeric monomer mixture may be the cause for an increase in dispersity, as the isomers differ in their reactivity.^[^ ^48^ ^]^ For instance, it has been demonstrated that mixing of pure *cis*‐1,3‐pentadiene with *trans*‐1,3‐pentadiene affords broad distributions (Figure 1a), while polymerization of pure *trans*‐1,3‐pentadiene gives low dispersity (*Ɖ* = 1.09). This indicates the significant influence of the stereochemistry on the chain growth process.^[^ ^49^ ^]^ However, the presence of a high content of the *trans*‐isomer may potentially induce aggregation of the thioether moiety with the counter ion, resulting in broadening of the molecular weight distributions. Similar to the reported isomers, we observed peculiar tailing to higher elution volume, hinting at complex polymerization kinetics.

**Table 1:** Summary of synthesized homopolymer PMTHD and copolymers PMTHD‐*co*‐PI.

Entry	Polymer	*M*_n,theo_ (kg mol^−1^)	*x* ^MTHD^	*x* ^Isoprene^	*w* ^MTHD^	Solvent	*M*_n,SEC_ ^a)^ (kg mol^−1^)	*Ɖ* ^a)^	*t* (h)	*T*_g_ ^b)^ (°C)
1	PMTHD_34_	5	1	0	1	CHx	4.3	1.34	5	−34
2	PMTHD_66_	10	1	0	1	CHx	8.4	1.35	6	−32
3	PMTHD_133_	20	1	0	1	CHx	17.0	1.24	6	−30
4	PMTHD_299_	50	1	0	1	CHx	38.3	1.28	6	−25
5	PMTHD_31_	5	1	0	1	MTBE	4.0	1.29	7	−36
6	PMTHD_68_	10	1	0	1	MTBE	8.7	1.21	7	−32
7	PMTHD_90_	20	1	0	1	MTBE	11.5	1.39	7	−30
8	PMTHD_6_‐*co*‐PI_202_	10	0.05	0.95	0.09	CHx	14.5	1.04	8	−62
9	PMTHD_11_‐*co*‐PI_181_	10	0.10	0.90	0.17	CHx	13.7	1.05	8	−62
10	PMTHD_3_‐*co*‐PI_308_	20	0.02	0.98	0.04	CHx	21.4	1.04	7	−63
11	PMTHD_8_‐*co*‐PI_275_	20	0.05	0.95	0.09	CHx	19.7	1.06	7	−62
12	PMTHD_18_‐*co*‐PI_301_	20	0.10	0.90	0.17	CHx	22.8	1.07	7	−59
13	PMTHD_46_‐*co*‐PI_782_	50	0.10	0.90	0.17	CHx	59.2	1.19	8	−61

^a)^ Determination via a SEC (Eluent: THF; 30 °C) utilizing a PI‐standard.

^b)^ Determination via DSC measurements, based on second heating curve.

In another series with molar masses up to 20 kg mol^−1^, we increased the solvents’ polarity by use of methyl *tert*‐butyl ether (MTBE) at room temperature (Figures 1b, S12). MTBE is suitable for the anionic polymerization and is less prone to proton abstraction compared to the established polar solvent THF.^[^ ^50^ ^]^ MTBE resulted in improved solubility of the polymethylthiohexadienyl chain ends. However, the resulting homopolymers still exhibited slightly elevated dispersities, with *Ɖ* values exceeding 1.21 (Figure 1b). The respective ^1^H NMR spectrum reveals no notable influence on the polydiene micro‐structure (Figure S13).

The glass temperatures of PMTHD have been determined via DSC measurements (Figure S14). Extrapolation of the inverse molar masses and the obtained glass temperature gave the respective value for *T*_g,∞_ = −26 °C (Figure S15). This high chain flexibility is a first indication for the regiostructure (see below). The structurally related polybutadiene exhibits this increased *T*_g_ only in the case of high content of vinyl side chains. The microstructure was investigated in a detailed manner by NMR, as discussed below.

Microstructure of PMTHD

The regiostructure of PMTHD was determined using combined NMR spectroscopy methods and referring to reports on structurally related polydienes.^[^ ^48^, ^51^ ^]^ Figures S8–S12 present the ^1^H NMR, ^1^H−^1^H COSY, ^13^C−DEPT, ^1^H−^13^C HSQC and ^1^H−^13^C HMBC spectra. The ^1^H NMR spectrum (Figure S8) provides only evidence of 1,4‐ and 1,2‐PMTHD, with no indication of vinyl protons, which would be observed in a 3,4‐regiostructure. Nonetheless, the broad signal indicates no uniform microstructure, resulting in strong signal overlap. Additionally, increased line width could be attributed to a short T2 time, which prevents visualization of existing couplings in 2D NMR experiments. However, ^1^H−^13^C HSQC and ^1^H−^13^C HMBC gave valuable information for the assignment of the respective protons and carbons in both 1,2‐ and 1,4‐PMTHD. Prior to the HSQC and HMBC measurements (shown below), ^13^C DEPT measurements were utilized to differentiate between the methyl, methylene and methine groups. Relying on the HSQC and HMBC spectra, the signals of the side chains could be identified. Both the methylene (5, 5′) as well as the methyl carbons (7, 7′) show two separate signals, indicating the presence of both 1,4‐ and 1,2‐microstructures. The relative intensities of the respective signals in the ^13^C NMR spectrum suggest that their abundance is almost equal. No separate signals of both microstructures are present in the ^1^H NMR spectrum. As a result, the exact ratio could not be determined.

These findings indicate a significant amount of 1,2‐microstructure, which translates to a different mechanism compared to the anionic polymerization of butadiene or isoprene. As observed recently for other monomers, oxygen atoms in the side chain caused the so‐called “self‐modification”, that is, coordination to the lithium‐counterion explains the increase in 3,4‐units.^[^ ^37^, ^38^ ^]^ Compared to oxygen, the electrons of sulfur in MTHD are less localized. Therefore, weaker coordination of the “soft” sulfur atom to the hard lithium counterion is expected.

### In Situ NMR Kinetics of MTHD

The broad distributions obtained from the anionic polymerization of the isomeric mixture of MTHD may be due to unequal reactivity of both isomers, as already reported for the anionic polymerization of *cis*‐ and *trans*‐ocimene.^[^ ^48^ ^]^ *Cis*‐ and *trans*‐MTHD show a significant separation of their respective olefinic signals in the ^1^H NMR spectrum (Figure S2). Therefore, in situ ^1^H NMR kinetics was conducted for MTHD polymerization in C_6_D_12_, tracking the integrals of the C2 vinyl protons of the *cis* (δ = 6.70 – 6.56 ppm) and *trans* (δ = 6.37 – 6.20 ppm) isomers over time, highlighted in Figure 1c. As indicated in Figure S16a, a rapid polymerization with almost full consumption of the *cis*‐MTHD occurred already after 4 min, while traces of *trans*‐MTHD were still present after 12 min. However, *trans*‐MTHD was present in excess over the *cis*‐isomer. The reactivity ratios were evaluated with a non‐terminal model (*r*_1_ *r*_2_ = 1; Jaacks^[^ ^52^ ^]^), yielding the values *r_cis_* = 2.75 and *r_trans_* = 0.364, translating to favorable incorporation of the *cis*‐MTHD. In case of ocimene, which is the only other isomeric 1,3‐diene investigated via the same methodology to date, the online NMR study demonstrated favorable incorporation of the *trans*‐ocimene.^[^ ^48^ ^]^ However, ocimene is a disubstituted diene monomer. A more significant comparison can be made with work published on 1,3‐pentadiene. Here, the isomeric mixture of the 1‐subsituted isomeric mixture exhibited rate constants in the order *cis* > *trans*, which were separately determined in toluene.^[^ ^53^ ^]^ The following experiments deliberately neglect the presence of *cis*‐MTHD to simplify the discussion. Nevertheless, despite the small fraction in the isomeric mixture and quantitative reaction within a relatively short time, its presence should not be ignored.

### Copolymerization of MTHD with Isoprene

In contrast to the vulcanization process, in which polydienes are crosslinked with sulfur and additives as curing agents, originally invented by Charles Goodyear,^[^ ^54^ ^]^ our objective was to achieve a distinct incorporation of sulfur in the polydiene chains by anionic copolymerization of MTHD with isoprene. To prevent a major influence on the excellent elastic properties of polyisoprene, the MTHD content, *x* ^MTHD^, was kept low with values varying from 2% to 10%, with targeted molar masses between 10 and 50 kg mol^−1^. The obtained copolymers were characterized regarding molar mass, dispersity and glass temperatures, shown in Table 1 (entries 8−13).

**Figure 2:**
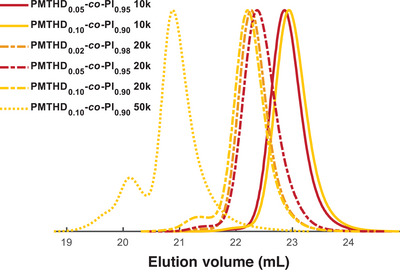
SEC traces (RI Signals, eluent: THF) of the copolymers of isoprene and MTHD, varying *x* ^MTHD^ and increasing *M*_n,theo_.

SEC traces (Figure 2) of lower molar mass PMTHD‐*co*‐PI samples are monomodal with low dispersities (*Ɖ* < 1.07), indicating good control over the copolymerization regardless of *x* ^MTHD^. Only the copolymer with a targeted molar mass of 50 kg mol^−1^ exhibited slight bimodality, attributed to oxygen coupling introduced due to insufficient degassing of methanol utilized for termination. This resulted in an increase in dispersity (*Ɖ* = 1.19). As shown in Figure S18, ^1^H NMR spectroscopy confirms the molar amount *x* ^MTHD^. DSC characterization of all copolymers revealed low glass temperatures <−59 °C (Figure S19). Consequently, the thermal properties of PI are not changed by the presence of up to 10 mol% of MTHD, making the copolymers suitable for elastomer applications.

### In Situ NMR Kinetics of Copolymerization of MTHD and Isoprene

**Figure 3:**
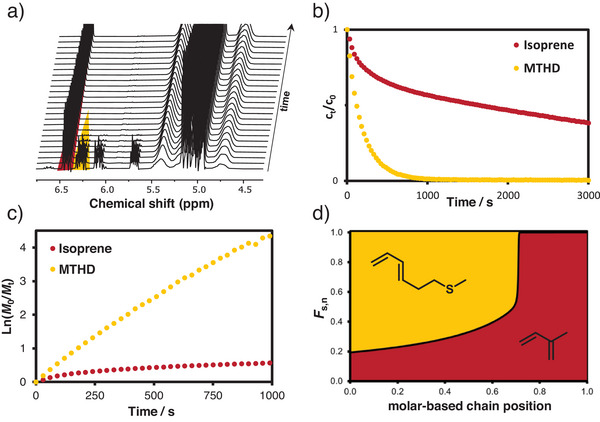
a) Stacked ^1^H NMR spectra of the copolymerization of MTHD and isoprene, b) individual conversion plots of MTHD and isoprene over time, c) pseudo‐first‐order plot for the polymerization of PMTHD‐*co*‐PI and d) molar composition profile of an equimolar monomer mixture derived from the calculated reactivity ratios.

For any application, the distribution of MTHD within the copolymer chains is relevant. Therefore, in situ NMR kinetics studies were carried out in C_6_D_12_. (details: Supp. Inf.). As emphasized in Figure 3a, the signals of isoprene (red, δ = 6.48–6.38 ppm) and *trans*‐MTHD (orange, δ = 6.34–6.21 ppm) were baseline‐separated, permitting to track the individual monomer consumption over time. The in situ NMR data revealed preferential consumption of *trans*‐MTHD over isoprene (Figure 3b), suggesting fast crossover reaction from polyisoprenyl chain ends to MTHD, resulting in a pronounced gradient structure (Figure 3d). The reactivity ratios of MTHD and isoprene were *r*_MTHD_ = 3.2 and *r*_I_ = 0.0027. In this case, the logarithmic Meyer‐Lowry fit was utilized to determine the reactivity ratios, as this method gave theoretical values in good agreement with the obtained data (Figure S20). The known reactivity ratios of isoprene and butadiene *r*_I_ = 0.42 and *r*_B_ = 2.82^[^ ^55^ ^]^ suggest a weaker gradient for copolymerization of MTHD and the industrially established butadiene, which will be studied in future work.

### Post‐Polymerization Modification

Following the successful incorporation of thioether moieties in the copolymers, we studied post‐modification of the thioether side chains. We investigated oxidation, alkylation and alkoxylation.^[^ ^10^, ^14^, ^15^, ^16^, ^17^, ^18^, ^19^, ^20^, ^21^, ^22^, ^23^ ^]^ These reactions can be utilized to alter polymer properties, for example by increasing polarity or introducing polyelectrolyte characteristics.

**Figure 4:**
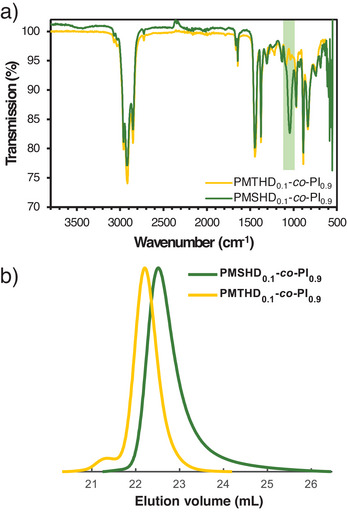
Comparison of the oxidized copolymer (green) and the respective precursor (yellow) in a) IR spectra permitting to identify the stretching vibration of the formed sulfoxide at 1040 cm^−1^ (highlighted in green) and b) SEC measurements to identify the expected shift in molar mass.

Oxidation of the thioether to the sulfoxide strongly increases the dipole moment and polar character. However, oxidation of polydienes has been reported to convert the double bonds of the polymer backbone to epoxides.^[^ ^56^, ^57^ ^]^ For the MTHD copolymers, selective oxidation of the thioether moiety was targeted, without affecting the polydiene backbone. Selective oxidation of the sulfide to sulfoxide was accomplished using *m*‐chlorobenzoic peroxide in DCM, keeping reaction times short.^[^ ^58^ ^]^ The MTHD repeating units of the copolymer are converted to 6‐methylsulfinyl hexadiene (MSHD). The formed sulfoxide was identified via FT‐IR‐spectroscopy (Figure 4a), evidenced by the respective band at 1040 cm^−1^, assigned to the S═O double bond. Concurrently, the C═C double bonds remained unaltered, thereby demonstrating their stability under the chosen conditions (Figure S21). Undesired oxidation of the double bonds is a major disadvantage for the application of polydienes. The polydiene maintained its low *T*_g_ = −58 °C, as revealed by DSC measurements (Figure S25).

#### Alkoxylation

**Scheme 2:**
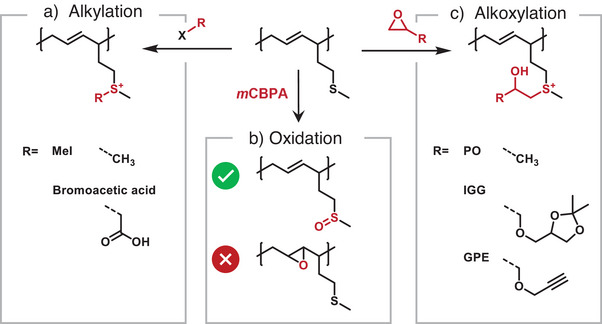
Overview of post‐polymerization modification of thioether moieties. a) Alkylation with methyl iodide and bromoacetic acid, b) oxidations and c) alkoxylation with the three epoxides exemplary used (PO, IGG and GPE).

**Figure 5:**
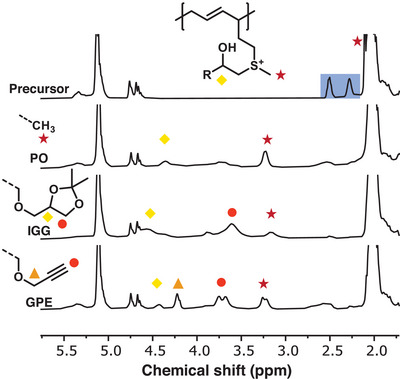
Stacked ^1^H NMR spectra (400 MHz, CDCl_3_) of the alkoxylation reaction with either PO, IGG or GPE in comparison to the precursor copolymer PI_0.9_‐*co*‐PMTHD_0.1_ (methyl group indicated with a red star and methylene group of thioether highlighted in blue).

Deming and coworkers reported the successful addition of epoxides at thioether groups in polypeptides based on l‐methionine.^[^ ^15^ ^]^ A variety of functional epoxides and glycidyl ethers, respectively, were utilized to address the thioether moiety in poly(l‐methionine). Soon after, thioether groups were also introduced in polyethers and polycarbonates, respectively.^[^ ^16^, ^21^ ^]^ In all cases, the reactivity of the sulfur (II) species was successfully manipulated by the pH value. At low pH the sulfide attacks the activated epoxide ring. Inspired by these works, this functionalization was studied with the copolymer PI‐*co*‐PMTHD (Table 1, entry 12). Three exemplary epoxides were chosen for polymer modification (Scheme 2). In addition to industrially highly‐established propylene oxide (PO), we selected 4‐[(2,3‐epoxypropoxy) methyl]‐2,2‐dimethyl‐1,3‐dioxolane (IGG) and glycidyl propargyl ether (GPE) due to their functional groups. We expected that the acidic conditions of the alkoxylation would cleave the acetal group of IGG to form two additional hydroxyl groups. The respective ^1^H NMR spectra (Figure 5) illustrate full conversion of the thioether group. The signal of the methylene group (red stars) broadens and shifts from 2.1 to around 3.2 ppm. Additional signals arise that depend on the epoxide employed. FT‐IR further supports the formation of hydroxyl groups with the characteristic broad band (Figures S23–S25). DSC measurements were conducted as well to investigate the influence of the functionalities attached for the glass temperature. As illustrated in Figure S26, for all three samples containing 10 mol% of modified MTHD units, the polydiene low glass temperatures below −60 °C were measured.

#### Alkylation

In analogy to the alkoxylation, due to the high nucleophilicity of thioethers compared to ethers, alkylation enables access to highly functional sulfonium derivatives.^[^ ^14^ ^]^ This reaction was utilized by Long et al. and Matyjaszewski and coworkers for the bio‐inspired 2‐(methylthio)‐ethyl methacrylate to generate polymer electrolytes complexing siRNA and DNA, respectively.^[^ ^18^, ^20^ ^]^ Here, methyl iodide and 2‐bromoacetic acid were selected, as demonstrated in Scheme 2. ^1^H NMR spectroscopy (Figure S27) confirms high conversion of the thioether, indicated by decreasing signals of the neighboring methylene groups (δ = 2.6–2.2 ppm). New signals arise in both cases that can be assigned to the methyl group attached to the sulfur‐atom (Figures S28 and S29). FT‐IR spectroscopy of the carboxylated copolymer shows new bands that are assigned to the carboxyl group (1628 cm^−1^). DSC measurements showed no change of the glass temperature for the methylated copolymer with a *T*_g_ = −59 °C. A slight increase with a *T*_g_ = −57 °C was found for the carboxylated PI‐*co*‐PMTHD (Figure S30).

### Evaluation of Inhibition of Bacterial Growth

**Figure 6:**
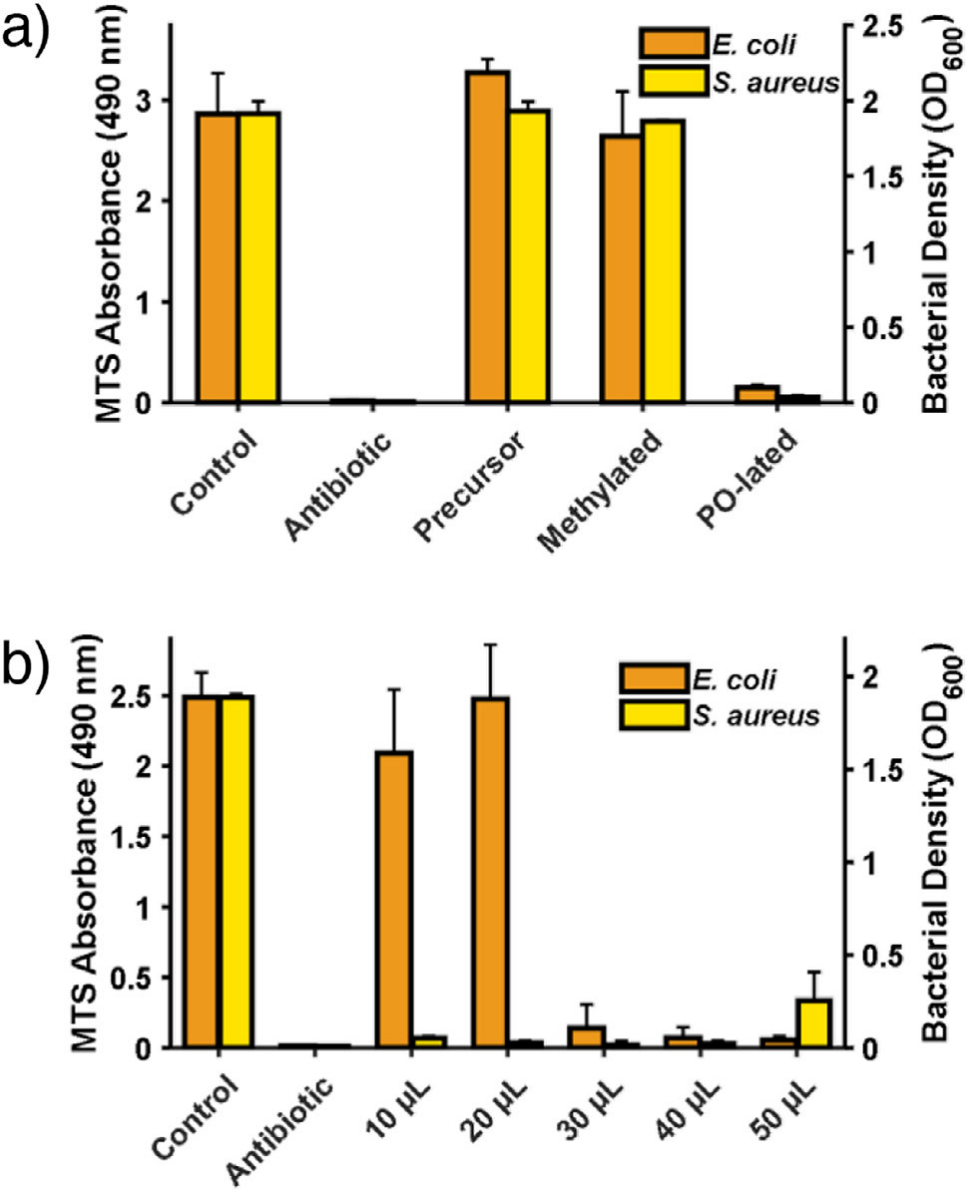
Bacterial growth via MTS absorbance for *E. coli* (MTS assay, orange) and via the bacterial density for *S. aureus* (OD_600_, yellow). a) Different chemical modification of PMTHD_0.1_‐*co*‐PI_0.9_ of 50 µL 1 mL^−1^ aliquots and b) increasing volume fraction of the PO‐lated PMTHD_0.1_‐*co*‐PI_0.9_ in the total 1 mL sample volume. In both cases, measurements are referred to the antibiotics ampicillin (*E. coli*) and kanamycin (*S. aureus*). Error bars indicate the standard deviation of the data.

Sulfonium groups are known as effective antimicrobial moieties, with demonstrated efficacy against various bacterial strains. This effect is attributed to the anionic microbial membrane, particularly in gram‐negative bacteria, which can be targeted passively. This ultimately leads to a disruption of the bacterial membrane. However, in contrast to the typically utilized nitrogen‐containing moieties, sulfonium groups show high activity against gram‐positive strains.^[^ ^3^, ^11^, ^12^, ^59^ ^]^ To obtain preliminary insights, we sought to examine the performance of the copolymer PMTHD_0.1_‐*co*‐PI_0.9_ (Table 1, entry 12) with tertiary sulfonium species as an antimicrobial coating. We selected the PO‐functionalized and the methylated samples as typical representatives for both alkoxylation and alkylation. MTS assays were carried out to quantify the inhibitory effect of the modified polymers on the growth of *Escherichia coli* (*E. coli*) strains (Figure 6). Following the coating of the polymer stock solution to various surfaces, *E. coli* was introduced and incubated overnight. Remarkably, the sample modified with propylene oxide demonstrated a strong inhibitory effect on bacterial growth, even comparable to that observed with the antibiotic ampicillin. In comparison, modification with methyl iodide did not show signs of inhibition under the chosen conditions. Also, the precursor copolymer does not affect bacterial growth, proving the high impact of modification with propylene oxide. Furthermore, we were able to identify the concentration dependency for the PO‐transformed copolymer (Figure 6b).

Moreover, the growth of gram‐positive *Staphylococcus aureus* (*S. aureus*) on various surfaces was examined. As with the observations made for *E. coli*, the copolymer modified with propylene oxide demonstrated strong inhibitory effects on bacterial growth, comparable to those observed with the common antibiotic kanamycin (Figure 6). Subsequent experiments conducted with decreasing concentrations of the PO‐lated copolymer, demonstrated enhanced performance against *S. aureus*. As illustrated in Figure S32, a notable effect can be achieved with a mere sixth of the volume of the copolymer‐containing solution. This enhanced potency of sulfonium species against gram‐positive over gram‐negative strains has already been described by Hirayama et al.^[^ ^60^ ^]^ The elevated bacterial density observed for the setup with the highest copolymer concentration (Figure 6) is presumably attributed to aggregation. This is supported by the fact that the microbial activity in Figure S33 exhibits low values, comparable to those observed for the lower concentrations.

We hypothesize that modification of the thioether with glycidyl ethers with functional groups known for their antimicrobial impact (e.g., ammonium and guanidine) could further improve the performance.

## Conclusion

Synthesis and anionic polymerization of the first thioether functional diene monomer, 6‐methylthio hexa‐1,3‐diene (MTHD) have been described. A straightforward synthesis requiring only a single purification step was accomplished, resulting in preferential formation of the *trans*‐isomer. The anionic homopolymerization with targeted molar masses of *M*_n_^targ^ = 5–50 kg mol^−1^ resulted in increased dispersities of *Ɖ* > 1.24, tentatively explained by the presence of an isomeric monomer mixture. In situ ^1^H NMR kinetics revealed different reactivities of the *cis*‐ and the *trans*‐MTHD isomer. Copolymerization with isoprene resulted in well‐defined structures with dispersities of *Ɖ* < 1.19. The molar composition of the copolymers with molar MTHD fractions of *x* ^MTHD^ = 2%−10% was further examined via ^1^H NMR kinetics, revealing moderate gradients (*r*_MTHD_ = 3.2 and *r*_I_ = 0.0027). We further established various post‐polymerization functionalizations based on PI‐*co*‐PMTHD copolymer. Alkoxylation was demonstrated, using epoxides with methyl‐, propargyl‐ and glyceryl groups. Additionally, alkylation reactions with methyl iodide and bromoacetic acid were conducted. Finally, the thioether group was selectively oxidized to the corresponding sulfoxide. All post‐polymerization modifications change the solubility of the copolymers considerably, while not strongly affecting the *T*_g_. Moreover, the modification with propylene oxide induced antimicrobial properties with high performance against *S. aureus* strains. In addition, the selectivity of the oxidation leads to the hypothesis that MTHD might serve as an internal antioxidant for elastomer applications. Furthermore, MTHD paves the way to a new class of sulfur containing materials, as it represents a platform for the fabrication of antimicrobial surfaces.

## Supporting Information

The Supporting Information is available free of charge, with descriptions of experimental methodology (i.e., monomer synthesis, polymer synthesis, kinetics) and data on NMR characterizations, calorimetric measurements, evaluation of bacterial activity.

## Conflict of Interests

The authors declare no conflict of interest.

## Supporting information



Supporting Information

## Data Availability

The data that support the findings of this study are available in the Supporting Information of this article.
