# Supplementary material for: Methylthiohexa‐1,3‐Diene: Anionic Polymerization of a Diene with Thioether Moiety Enables Post‐Polymerization Modification and Antimicrobial Materials
Source: Angew Chem Int Ed Engl. 2025 Aug 10;64(39):e202508129. doi: 10.1002/anie.202508129 (PMC12455420; doi:10.1002/anie.202508129)
Supplement: Supplementary file 1 — Supporting Information [file ANIE-64-e202508129-s001.docx]

**Supporting Information**

Methylthiohexa-1,3-diene: Anionic Polymerization of a Diene with Thioether Moiety Enables Post-Polymerization Modification and Anti-microbial Materials

Moritz Rauschenbach^[a]^, Matthias Bros^[b]^, Holger Frey *^,[a]^

^a^Department of Chemistry, Johannes Gutenberg-University Mainz, Duesbergweg 10−14, D‑55128 Mainz, Germany

^b^ University Medical Centre, Johannes Gutenberg University, Langenbeckstraße 1, D-55101 Mainz, Germany

Instrumentation

**Gel Permeation Chromatography (SEC)**

SEC analysis was conducted utilizing an Agilent 1260 Infinity II instrument. It is equipped with a MZ-GEL-DS plus 10^5^/10^3^/100 Å column set from MZ-Analysetechnik (Mainz, Germany) and a RI detector (Agilent G1362A) was used for every detection. The eluent used was THF, with an injection volume of 100 µL. The columns were heated to 30°C and a flow rate of 1 mL/min was used for all measurements. Calibrated was performed using a toluene standard, and polyisoprene standards were sourced from PSS Polymer Standard Service GmbH (Mainz, Germany).

**Nuclear Magnetic Resonance (NMR) spectroscopy**

^1^H, ^13^C, ^1^H−^1^H-COSY, ^1^H−^13^C-HSQC, ^1^H−^13^C-HMBC NMR spectra were recorded on a Bruker Avance III HD 300, Bruker Avance II HD 400 and Bruker Avance III 600 spectrometer. *In situ* NMR kinetics were investigated on a Bruker Avance III HD 400 spectrometer. All spectrometers are equipped with a 5 mm BBFO-SmartProbe with z-gradient and ATM as well as a SampleXPress 60 sample changer. The signals were referenced internally to the assigned proton signal of the used deuterated solvent (CDCl_3_, DMSO-*d*^6^ and C_6_D_12_). All spectra were evaluated using MestReNova 14.2.0 developed by Mestrelab Research S.L. (Santiago de Compostela, Spain).

**Differential Scanning Chromatography (DSC)**

The thermal properties were investigated with a DSC 250 from TA instruments with an RCS 90 compressor. Calibration was achieved with an indium and *n*-octane standard. Prior to the measurements the polymers were dried under high vacuum. At least 5 mg polymer was placed into a subsequently sealed pan. Any thermal history of the samples was removed through heating to 120°C. Glass temperature values were extracted from the second heating ramp starting from ‑90 °C to 120°C using a rate of 10 K/min. All measurements were performed under a nitrogen atmosphere.

**Fourier-Transformed Infrared-Spectroscopy**

FT-IR spectroscopy was conducted on a diamond ATR unit-equipped *Nicolet* iS10 FT-IR spectrometer (Thermo Scientific, Waltham, MA, USA).

Experimental Section

**Reagents**

All reagents were purchased from commercial suppliers. Methional was received from Sigma Aldrich. We further purchased from Fisher Scientific, TCI and Acros Organics. Chloroform-*d*, dimethyl sulfoxide-*d*^6^ and cyclohexane-*d*^12^ were purchased from Deutero GmbH. All reagents were used as received if not stated otherwise. Methional was distilled prior to use. Cyclohexane was dried before use with sodium and benzophenone as an indicator while MTBE was purified with *s*BuLi and DPE as an indicator.

**Monomer Synthesis.**

6-Methylthiohexa-1,3-diene was synthesized in a three-step synthesis.

1-Methylthiohexa-5-en-3-ol was prepared in a Grignard reaction starting from methional. In three-necked round-bottom flask Mg-turnings (6.3 g; 0.26 mol) were suspended in 40 mL anhydrous diethyl ether. A solution of 24.3 mL (0.28 mol) in 180 mL was added dropwise so that the suspension was mildly boiling. Once all the allyl bromide was added the solution was stirred for an additional hour. In another three-necked round-bottom methional (20 mL; 0.2 mol) was dissolved in 50 mL anhydrous diethyl ether and cooled in an ice-bath. To the cooled solution the freshly prepared Grignard-solution was added dropwise. Upon complete addition, the solution was stirred for an additional 30 minutes before warmed up to room temperature. The reaction was quenched by the addition of 200 mL of a saturated NH_4_Cl-solution. The aqueous phase was extracted with 80 mL of diethyl ether. The combined organic extracts were washed two times with 100 mL of water and one time with 100 mL brine before dried over Na_2_SO_4_. The solvent was removed under reduced pressure to yield 22.7 g (77%) of the crude alcohol which was used without further purification.

The crude 1-methylthiohexa-5-en-3-ol was converted into the respective tosylate was adapted from Yokochi *et al*.^1^ 21.5 g (0.15 mol) of 1-methylthiohexa-5-en-3-ol, 41.0 mL (0.29 mol) triethyl amine and 19.8 g (0.16 mol) DMAP were dissolved in dichloromethane (130 mL) and cooled to 0 °C. 46.5 g (0.24 mol) of *p*-toluenesulfonyl chloride was dissolved in 50 mL DCM and was added dropwise to the solution. The mixture was allowed to heat to room temperature before stirred for 2 hours. The solution got poured in 200 mL saturated ammonium chloride solution. The mixture was extracted 3 times with 70 mL of diethyl ether. The etheric solutions were washed with 100 mL of 1 m HCL, 2 times 100 mL H_2_O and with 100 mL of brine. After drying over Na_2_SO_4_ the solvent was removed under reduced pressure. Without further purification the compound was used.

The synthesis of the 6-methylthiohexa−1,3-diene started by dissolving the tosylate in 180 mL toluene. 36.8 mL (0.59 mol) DBU was added, and the solution was heated to 100 °C overnight. After cooling to room temperature 60 mL of saturated ammonium chloride solution, 60 mL deionized water and 80 mL diethyl ether were added. The product was extracted three times with diethyl ether. The combined organic extracts were washed with 1 m HCl-solution (2 x 50 mL), water (60 mL) and brine (60 mL). The solvent was removed under reduced pressure. The product was purified by fractionated distillation at 30 mbar to yield 9.23 g of MTHD as a colorless liquid (50%).

**^1^H NMR** (CDCl_3_; 300 MHz): δ[ppm]= 6.65 (dddd, H-2*^cis^*), 6.34 (dt, H-2*^trans^*, 1H), 6.20 – 6.03 (m, H-3, 1H), 5.80 – 5.69 (m, H-4, 1H), 5.20 – 4.99 (m, H−1, 2H), 2.59 (ddd, H-6, 3H), 2.41 (q, H-5, 2H), 2.14 (s, H-7, 3H).

**^13^C NMR** (CDCl_3_; 101 MHz): δ[ppm] = 136.89 (C-2*^trans^*), 132.70 (C-4*^trans^*), 132.24 (C-3*^trans^*), 131.88 (C-2*^cis^*), 130.49 (C-4*^cis^*), 130.09 (C-3*^cis^*), 117.83 (C−1*^cis^*), 115.76 (C−1*^trans^*), 34.11 (C-6), 32.34 (C-5), 15.55 (C-7).

**Table S1**: Tested conditions for the elimination of the tosylate to yield the highest ratio of *cis*/*trans* on a small scale. Values might change when upscaled.

| Entry | Solvent | Base | *T*  °C | *trans*^a^  % | *cis*^a^  % | Conversion. after 1h^a^ |
| --- | --- | --- | --- | --- | --- | --- |
| 1 | DMSO | KOtBu | 75 | 67 | 37 | >99% |
| 2 | DMSO | KOtBu | 35 | 77 | 23 | >99% |
| 3 | DMF | KOtBu | 35 | 85 | 15 | >99% |
| 4 | THF | KOtBu | 35 | 80 | 20 | >99% |
| 5 | THF | KOH^b^ | 35 | 71 | 29 | <50% |
| a) determined via ^1^H NMR spectroscopy b) addition as a 4 m solution in MeOH | | | | | | |

**Alternative monomer synthesis was achieved using the Wittig reaction.**

In a Schlenk-flask 34.5 g (0.09 mol, 1.2 eq) allyl triphenyl phosphonium bromide was suspended in 140 mL anhydrous THF. 10.10 g (0.09 mol, 1.2 eq) potassium *tert*-butoxide was added and the suspension was stirred for 30 min at room temperature. Subsequently, 7.5 mL (0.075 mol) methional were added dropwise and the solution was stirred overnight. The reaction was quenched by the addition of 15 mL n-pentane. The solution was concentrated by removing THF at reduced pressure before the TPPO salts were removed via centrifugation. The residual solvent was removed at reduced pressure and 1.71 g (18%) MTHD was obtained through fractionated distillation (1.0 10^-3^ mbar) yielding a *trans*/*cis* ratio of 54:46.

**General Polymerization Procedure**

Both homo- and copolymerizations were conducted in an Argon-filled glovebox (*MBraun*, < 0.1 ppm O_2_, < 0.1 ppm H_2_O). Prior, the monomers were suspended in dried calcium hydride and degassed using the *freeze-pump* technique. After stirring overnight, the monomers were distilled in another flask equipped with a Teflon stop cock filled with trioctyl aluminium to remove residual traces of impurities. The next day, the monomers were distilled into an empty Teflon stop cock-equipped flask.

**Procedure for *in situ* NMR kinetics**

In analogy to general polymerization procedures, the monomers were dried before use. Deuterated cyclohexane was freeze-dried over CaH_2_ and stirred overnight. This was repeated twice before C_6_D_12_ was transferred to a flame-dried flask. Monomers and solvent were transferred into the glovebox. Subsequently, the volumetrically prepared solution was filled into a conventional NMR tube and sealed with a septum. After initiation, we recorded spectra with a preset frequencency. The decreasing integrals of the monomers were then evaluated with NIREVAL to obtain the reactivity ratios.^2^ For the stereo copolymerization, we selected one scan every 5 seconds and a temperature of 20 °C to obtain enough data points for the evaluation of this fast polymerization reaction. In case of copolymerization with isoprene, one scan every 30 seconds and a temperature of 25 °C was selected.

**General Procedure for Alkoxylation**

The alkoxylation method of Deming *et al*.^3^ was adjusted to the polydiene system. 700 mg of the copolymer PMTHD_0.1_-*co*-PI_0.9_ were dissolved in a mixture of 5 mL DCM and 2.7 mL acetic acid (8 eq per MTHD units). Subsequently, the epoxide (3 eq per MTHD unit) was added, and the solution was stirred for 48 hours at room temperature. The solution was quenched by the addition of 7 mL of deionized water. The organic phase was washed with 7 mL of a 0.5 m HCl solution and 2-3 times with 7 mL brine. The solvent was removed under reduced pressure and the crude polymer was dispersed in water. The alkylated polymer was purified via dialysis. Finally, the freeze drying gave the alkylated polymer (yield: 68-88 %).

**General Procedure for Alkylation**

The alkylation procedure was performed in accordance with the procedure reported by Boyer *et al*.^4^ 700 mg of the copolymer PMTHD_0.1_-*co*-PI_0.9_ were dissolved in 7 mL DCM, prior to the addition of the alkyl halogenide (10 eq per MTHD unit). After 24-48 h of stirring at room temperature, the reaction was quenched by the addition 7 mL water. The organic phase was washed with 2-3 times with 7 mL brine. The solvent was removed under reduced pressure and the crude polymer was dispersed in water. The alkylated polymer was purified via dialysis. Finally, the freeze drying gave the alkylated polymer (yield: 83-94 %).

**Procedure for Postpolymerization Oxidation**

500 mg of the copolymer PMTHD_0.1_-*co*-PI_0.9_ were dissolved in 6 mL DCM. To the ice-cooled solution 0.23 g (1.5 eq per MTHD unit) *m*CPBA was added. The mixture was stirred for 1 hour at 0 °C. The mixture was poured into 6 mL saturated NaHCO_3_ and then washed with water and brine. The solvent was removed under reduced pressure and the polymer was finally freeze-dried with benzene.

**Procedure for Determination of Antimicrobial Activity**

E. coli K12 strain NEB-5a (PMID: 27834703) was cultured overnight in Luria-Bertani (LB) medium (10 g/l tryptone, 5 g/l yeast extract and 10 g/l sodium chloride; all from Carl Roth, Karlsruhe, Germany). S. aureus strain USA300 (PMID: 16517273) was grown overnight in tryptic soy broth (17 g/l pancreatic digest of casein, 5 g sodium chloride, 3 g peptic digest of soybean, 2.5g glucose, 2.5g dipotassium phosphate). Bacterial liquid cultures were grown in a bacterial shaker at 37 °C and 140 rpm (MaxQ 4000; Thermo Fisher, Waltham, MA). On the next day, bacterial liquid cultures were diluted 1:1,000 into fresh media, and 1 ml aliquots were set up. Antibotic (NEB-5α: 50 µg/ml Ampicillin, USA300: 25 µg/ml Kanamycin) and polymers at various doses were added to triplicate cultures per condition. After overnight incubation, optical density (OD600) was measured using a spectrophotometer (V−1200; VWR, Radnor, PA). Metabolic activity was assayed by detecting NAP(D)H oxidase activity. To this end, each 100 µl of cell suspension was transferred per well of a 96 well plate, and 20 µl of detection reagent comprised of a tetrazolium compound (MTS; 3-(4,5-dimethylthiazol-2-yl)-5-(3-carboxymethoxyphenyl)-2-(4-sulfophenyl)-2H-tetrazolium, inner salt) and phenazine ethosulfate as an electron coupling reagent. Plates were incubated for 1-3 h at 37 °C to allow for formaon of formazan product, Then, absorbance at OD490 was recorded (Spark Multimode Microplate Reader, Männedorf, Switzerland).

Characterization Data: Monomer Synthesis of 6-Methylthiohexa-1,3-diene (MTHD)

**Figure S1**: Crude ^1^H NMR spectra (DMSO-*d*^6^, 300 MHz) of 1-methylthiohexa-5-en-3-ol.

**Figure S2**: ^1^H NMR spectrum (CDCl_3_, 400 MHz) of 6-methylthio hexa-1,3-diene.

**Figure S3**: ^13^C NMR spectrum (CDCl_3_, 101 MHz) of 6-methylthio hexa-1,3-diene.

**Figure S4**: ^1^H-^1^H-COSY NMR spectrum (CDCl_3_, 400 MHz) of 6-methylthio hexa-1,3-diene.

**Figure S5**: ^1^H−^13^C-HSQC NMR spectrum (CDCl_3_, 400MHz, 101 MHz) of 6-methylthio hexa-1,3-diene with the expansion of relevant parts of the spectrum.

**Figure S6**: ^1^H−^13^C-HMBC NMR spectrum (CDCl_3_, 400MHz, 101 MHz) of 6-methylthio hexa-1,3-diene.


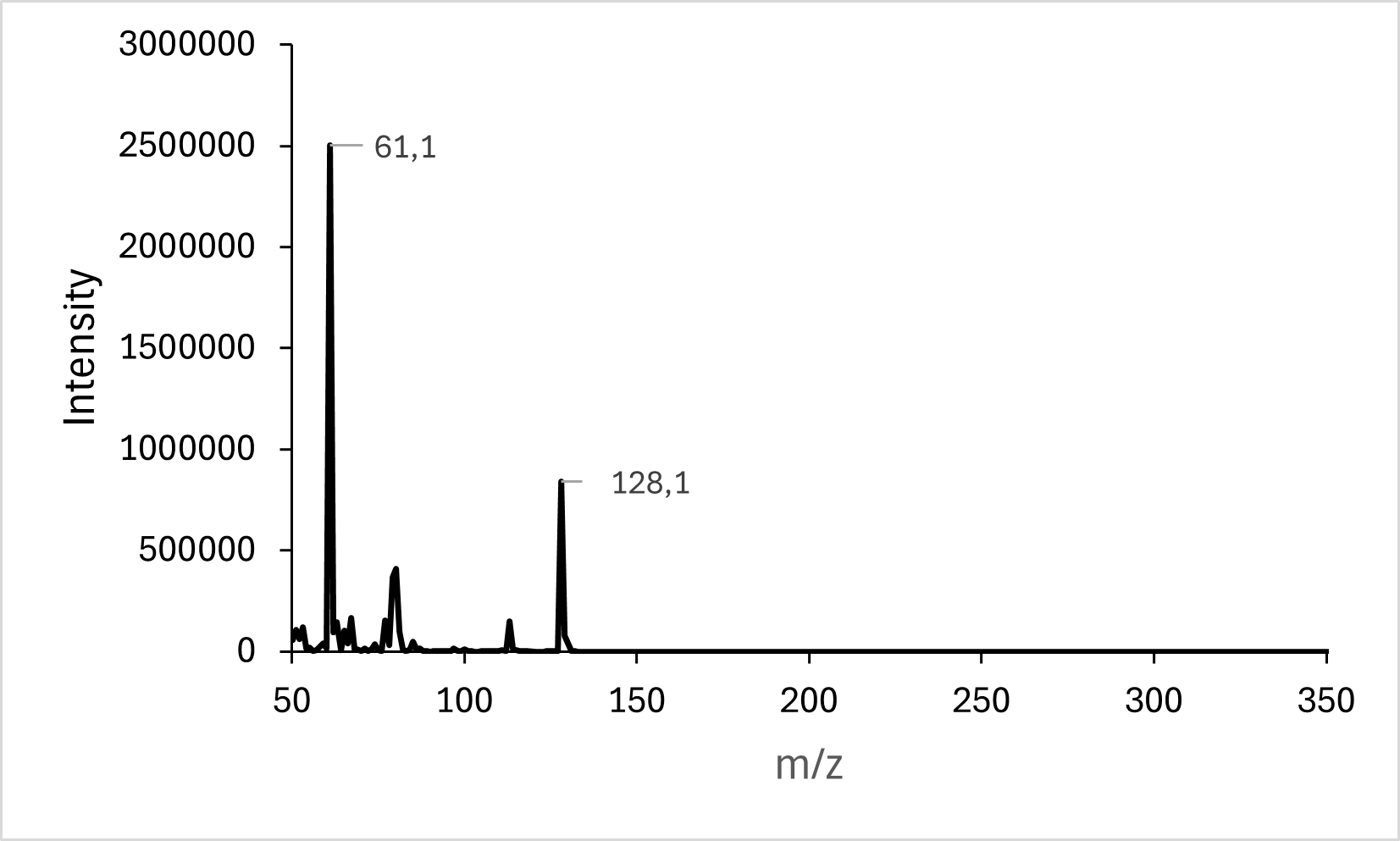


**Figure S7**: Gas chromatography mass spectrum of 6-methylthio hexa-1,3-diene (128,1 g mol^-1^) which was conducted on a Agilent GC 6850 equipped with a Agilent 5973 mass selective detector at 70eV. For the measurement a injection volume of 1 µL was purified in a RTX-5MS GC column. An additional signal was assigned to a fragmentation product CH_3_-S-CH_2_^+^ and the residual signals also belong to a fragmentation process initiated by chosen conditions of the analysis.

Characterization Data for Homopolymers PMTHD

**Figure S8**: ^1^H NMR spectrum (CDCl_3_, 600 MHz) of the homopolymer PMTHD polymerized in CHx (**Table 1**, entry 1).

**Figure S9**: ^13^C, ^13^C DEPT 90 and ^13^C DEPT 135 spectra (CDCl_3_, 101 MHz) of the homopolymer PMTHD (**Table 1**, entry 1).

**Figure S10**: ^1^H−^1^H COSY spectrum (CDCl_3_, 600 MHz) of the homopolymer PMTHD of the homopolymer PMTHD (**Table 1**, entry 1).

**Figure S11**: ^1^H−^13^C HSQC spectrum (CDCl_3_, 600 MHz) of the homopolymer PMTHD of the homopolymer PMTHD (**Table 1**, entry 1).

**Figure S12**: ^1^H−^13^C HMBC spectrum (CDCl_3_, 600 MHz) of the homopolymer PMTHD of the homopolymer PMTHD (**Table 1**, entry 1).


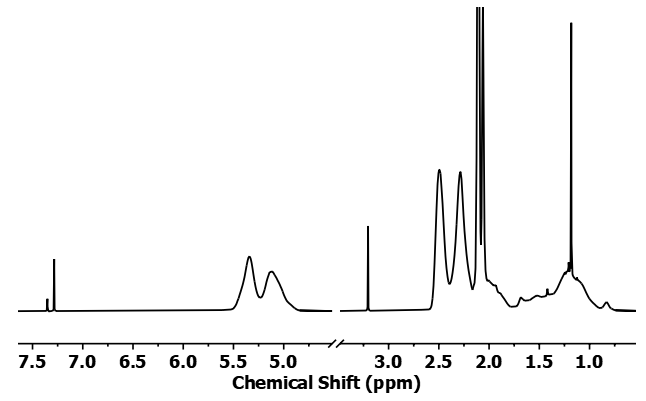


**Figure S13**: ^1^H NMR spectrum (CDCl_3_, 400 MHz) of the homopolymer PMTHD polymerized in MTBE (**Table 1**, entry 6).

**Figure S14** DSC curves of PMTHD polymerized in CHx (**Table 1**; entries 1-4).

**Figure S15**: Plot of glass temperature vs. the inverse molar mass for PMTHD (**Table 1**, Entries 1-4)


**Figure S16**: a) Monomer consumption over time of the two isomers of MTHD in the homopolymerization and b) the first-order plot of the *cis*- and *trans*-isomers of MTHD.

**Figure S17**: Plot of the individual monomer conversion versus the total monomer conversion of the stereo-copolymerization of *cis*- and *trans*-MTHD.

Characterization for Copolymers PI-*co*-PMTHD

**Figure S18**: Stacked NMR spectra of the copolymers PMTHD-*co*-PI (entries 10−12, **Table 1**) with a targeted molar mass of 20 kg mol^−1^. Increasing MTHD content is observable through the highlighted signals corresponding to MTHD.

******Figure S19**: DSC curves of the synthesized copolymers PMTHD-co-PI (**Table 1**; entries 8−13) with increasing molar mass.

**Figure S20**: a) Log Meyer-Lowry fit of the NMR kinetic experiment of the copolymerization of MTHD and isoprene and b) graphical illustration of the errors of the reactivity ratios.

Characterization Data for Oxidation Reaction

**Figure S21**: ^1^H NMR spectra (400 MHz, CDCl_3_) of the oxidized copolymer PI-*co*-PMSHD in comparison to the precursor PI-*co*-PMTHD.

**Figure S22**: DSC curve of the oxidized copolymer PI-*co*-PMSHD.

Characterization Data for Alkoxylation Reactions

**Figure S23**: IR spectra of the precursor PI-*co*-PMTHD (yellow) and the PO-functionalized copolymer (green).

**Figure S24**: IR spectra of the precursor PI-*co*-PMTHD (yellow) and the IGG-functionalized copolymer (green).

**Figure S25**: IR spectra of the precursor PI-*co*-PMTHD (yellow) and the GPE-functionalized copolymer (green).

**Figure S26**: DSC curves of the modified copolymer PI-*co*-PMTHD through alkoxylation with either PO, IGG or GPE.

Characterization Data for Alkylation Reactions

**Figure S27**: ^1^H NMR spectra (400 MHz, CDCl_3_) of the alkylated PI-*co*-PMTHD.

**Figure S28**: IR spectra of the precursor PI-*co*-PMTHD (yellow) and the methylated copolymer (green).

**Figure S29**: IR spectra of the precursor PI-*co*-PMTHD (yellow) and the carboxyl-functionalized copolymer (green).


**Figure S30**: DSC curves of the modified copolymer PI-*co*-PMTHD obtained via alkylation with methyl iodide or bromoacetic acid.

**Figure S31**: Visualization of the altered properties of the copolymer (middle) after the functionalization via alkylation with methyl iodide (left) and alkoxylation with propylene oxide (right).

Characterization of Antimicrobial Behavior

**Figure S32**: Illustration regarding bacterial density and metabolic activity of *S. aureus* bacterial strains on surfaces with decreasing concentration of the PO-lated copolymer PI_0.9_-*co*-PMTHD_0.1_ and the antibiotic Kanamycin as a reference.

**Figure S33**: Illustration of metabolic activity of *S. aureus* bacterial strains on surfaces with the highest concentrations of the PO-lated copolymer PI_0.9_-*co*-PMTHD_0.1_ and the antibiotic Kanamycin as a reference

References

1 P. R. Blakemore, S. K. Kim, V. K. Schulze, J. D. White and A. F. T. Yokochi, J Chem Soc Perkin 1, 2001, 0, 1831–1847.

2 M. Steube, T. Johann, M. Plank, S. Tjaberings, A. H. Gröschel, M. Gallei, H. Frey and A. H. E. Müller, Macromolecules, 2019, 52, 9299–9310.

3 E. G. Gharakhanian and T. J. Deming, Biomacromolecules, 2015, 16, 1802–1806.

4 P. Pham, S. Oliver, D. T. Nguyen and C. Boyer, Macromol Rapid Commun, 2022, 43, 2200377.
